# Pullulan-Based Hydrogels in Wound Healing and Skin Tissue Engineering Applications: A Review

**DOI:** 10.3390/ijms24054962

**Published:** 2023-03-04

**Authors:** Collins N. Elangwe, Svetlana N. Morozkina, Roman O. Olekhnovich, Victoria O. Polyakova, Alexander Krasichkov, Piotr K. Yablonskiy, Mayya V. Uspenskaya

**Affiliations:** 1 Chemical Engineering Center, ITMO University, Kronverkskiy Prospekt, 49A, 197101 Saint-Petersburg, Russia; 2 Saint Petersburg Research Institute of Phthisiopulmonology, Ligovsky Prospekt 2-4, 191036 Saint-Petersburg, Russia; 3 Department of Radio Engineering Systems, Electrotechnical University “LETI”, Prof. Popova Street 5F, 197022 Saint-Petersburg, Russia

**Keywords:** pullulan, chitosan, hydrogel, wound dressings, polysaccharides

## Abstract

Wound healing is a complex process of overlapping phases with the primary aim of the creation of new tissues and restoring their anatomical functions. Wound dressings are fabricated to protect the wound and accelerate the healing process. Biomaterials used to design dressing of wounds could be natural or synthetic as well as the combination of both materials. Polysaccharide polymers have been used to fabricate wound dressings. The applications of biopolymers, such as chitin, gelatin, pullulan, and chitosan, have greatly expanded in the biomedical field due to their non-toxic, antibacterial, biocompatible, hemostatic, and nonimmunogenic properties. Most of these polymers have been used in the form of foams, films, sponges, and fibers in drug carrier devices, skin tissue scaffolds, and wound dressings. Currently, special focus has been directed towards the fabrication of wound dressings based on synthesized hydrogels using natural polymers. The high-water retention capacity of hydrogels makes them potent candidates for wound dressings as they provide a moist environment in the wound and remove excess wound fluid, thereby accelerating wound healing. The incorporation of pullulan with different, naturally occurring polymers, such as chitosan, in wound dressings is currently attracting much attention due to the antimicrobial, antioxidant and nonimmunogenic properties. Despite the valuable properties of pullulan, it also has some limitations, such as poor mechanical properties and high cost. However, these properties are improved by blending it with different polymers. Additionally, more investigations are required to obtain pullulan derivatives with suitable properties in high quality wound dressings and tissue engineering applications. This review summarizes the properties and wound dressing applications of naturally occurring pullulan, then examines it in combination with other biocompatible polymers, such chitosan and gelatin, and discusses the facile approaches for oxidative modification of pullulan.

## 1. Introduction

The wound healing process is a complex and dynamic process of overlapping phases, and specific conditions are needed to support healing. The main goals of wound management are to provide a physical barrier against bacterial infections and to maintain an optimum moist environment, allowing the healing process to be accelerated [1,2,3]. The wound area must be covered with an ideal wound dressing in order to prevent the dressing from failing to function [4]. An ideal wound dressing material should have several specific properties, including: (a) biocompatibility; it is essential that the dressing not create any toxicity in the wound environment; (b) high absorption capacity of exudate; large amounts of wound fluids need to be removed, as wound exudates promote a high risk of bacterial colonies and growth; (c) adequate water vapor permeation rate; an optimal moisture level should be maintained in the wound, as a high level of water vapor permeation rate dehydrates the wound too quick and can cause scars, while a low level of water vapor permeation rate leads to excess exudates, thereby increasing the risk of bacterial infections; (d) good physical barrier; bacterial penetration must be prevented; (e) antimicrobial activity; microorganisms must not be able to grow under the dressing; and (f) non adhesive properties; adhesiveness of the wound dressing is most likely to increase the risk of further injuries upon removal [4,5,6]. Wound dressing materials are fabricated from naturally derived or synthetic polymers, or from a combination of the two. Wound covering materials are mostly produced as films, sponges, hydrocolloids, and hydrogels [7]. Until now, naturally derived polymers have received enormous attention in biomedical, pharmaceutical, and medical applications due to their biocompatibility and biodegradability properties [8]. There are still research challenges to developing multifunctional and cheap wound dressings through simple green synthesis approaches, as the dressings should show biocompatible, biodegradable, mucoadhesive, hemostatic, and bactericidal properties along with their main focus as wound dressings and drug delivery devices. Of the various types of wound dressings that have been fabricated, polysaccharide types have several advantages; for example, along with the above-mentioned properties, the hydrophilic groups on their polymers create a three-dimensional crosslinked network.

Naturally occurring polymers are greatly employed in the design and fabrication of wound dressing due to their similarity with the extracellular matrix (ECM) and nonimmunogenic properties that are detected with synthetic polymers [9,10]. Polysaccharides are a class of natural polymers made up of monosaccharide units and their derivatives [11]. Polysaccharides consisting of just one kind of monosaccharide unit are referred to as homopolysaccharides or homoglycans, while those containing more different types of monosaccharide units are called heteropolysaccharides or heteroglycans [12]. The main advantages of polysaccharides are their chemical properties; these properties are similar to heparin, providing the polymers with good hemocompatibility and making them less costly, in general, than other polymers [13]. Studies have shown that polysaccharides act as immunomodulatory materials to regulate inflammatory activities in wounds [14,15]. The main goal of this review is to discuss the properties and preparation of wound dressings from biopolymers that are based on polysaccharide pullulan which is incorporated with other polymers, such as chitosan and gelatin, as well as the facile approach of pullulan chemical modifications.

## 2. Types of Wound Dressing Materials

Dressing materials are generally classified depending on their activity and material origin. They are grouped into artificial, biomaterial, traditional, passive, and bioactive wound dressings [16]. Passive dressings offer physical barriers from the external surroundings and stop wound bleeding [17]. Traditional wound dressings, such as gauze, cotton, bandages, and gauze composites can absorb large volumes of wound fluids. However, traditional dressings can easily adhere to wound tissues, causing further damage of newly formed tissues and bleeding when removed. They also exhibit low vapor permeation properties. Wound fluids leaking out of these dressings can result in microbial contamination of the wound [18,19]. Biomaterial-based wound dressings can be categorized as allografts (skin substitutes), xenografts, or tissue derivatives [19]. Allografts are available either freeze-dried or newly supplied from donors. The application of an allograft is usually prevented by immune responses that lead to rejection by the recipient; they also carry a high risk of disease transmission and infection [20]. Moreover, allografts are quite expensive and have relatively low shelf-life [21]. Artificial wound dressings are available as films, gels, foams, and hydrocolloids [22,23]. Biopolymers are referred to as bioactive wound dressings; they include chitosan, alginate, cellulose, and gelatin, and are mostly used for their intrinsic and useful properties. Enhanced wound healing can be achieved by incorporating antibiotics, antioxidant, nanoparticles, and growth factors in the wound dressing [24,25,26,27]. Table 1 summarizes the various forms of wound dressings.

## 3. Properties of an Ideal Wound Dressings

Hydrogels synthesized from polysaccharides are known to be effective candidates for modern wound dressings. This is due to their large water-retention capacity, biocompatibility, non-toxicity, and biodegradability [32,33]. To date, several methods to design and fabricate efficient and cost-effective dressing materials have been investigated. Research has shown that wet dressings accelerated wound healing more than dry dressings. In the moist wound site only, there was healing, growth of new tissues, and re-epithelialization, with no occurrence of eschars or inflammation [34]. Hence, wet or moist wound dressings are the most preferred candidates for skin tissue repair, and hydrogel wound dressings are successful and effective because of their high-water content and permeability [35]. If a single fabricated wound dressing could possess most of these properties, then the wound healing process would be highly accelerated in a wet environment. With the increasing demand, fabrication of high performance modern wound dressings has become the main focus in research in the area of biomedical and pharmaceutical sciences, where hydrogels have been shown to fulfill most of the criteria for effective wound treatment. Hydrogels are a three-dimensional crosslinked network of hydrophilic polymeric materials [1] that can retain large volumes of water and swell without being dissolved. Hydrogels highly mimic the extracellular matrix of the skin, allowing them to be used extensively in biomedical fields. Hydrogels as wound dressing materials do not just offer physical barriers against microorganisms and absorb excess wound fluid; they can also effectively trap small biomolecules and provide an optimum moist surrounding which supports wound healing [32]. Properties of an ideal modern wound covering are shown in Table 2.

## 4. Bioactive Polysaccharide-Based Hydrogels

Ideal wound dressing materials should actively intervene in wound healing. Natural polysaccharides, such as chitosan and pullulan, have been used for preparing hydrogels in various biomedical applications. Chitosan is popular in tissue engineering applications due to its antimicrobial nature and biocompatibility [36]. Recently, pullulan has gained tremendous attention because of its unique properties and has been used in the fields of wound dressing and tissue engineering.

### 4.1. Chitosan

Chitosan is a naturally occurring homopolysaccharide and is the second most abundant macromolecule after cellulose. It is made up of β-D-glucosamine and N-acetyl-β-D-glucosamine units linked by 1,4-linkage, and its intrinsic properties, such as antimicrobial activity, low immunogenicity, non-toxicity, and biodegradability, have generated interest a great deal of interest [36]. These properties make chitosan a special material in wound dressing, delivery devices, and many other biomedical applications [37,38]. It is mainly obtained from the exoskeleton of crustaceans, such as waste from shellfish, shrimp, crabs, and lobsters [39,40] through incomplete deacetylation of chitin. Chitosan consists of amino and hydroxyl functional groups which permit it to react with other functionalized polymers through the formation of physical or chemical bonds. It is an antibacterial, biodegradable, and biocompatible polymer with high porosity and surface area, giving it wide applicability in the development of wound dressing materials and skin tissue scaffolds. Moreover, the presence of amino functional groups in the chitosan polymer facilitates the formation of complexes that include anionic macromolecules and nanoparticles through reactions with other cations. This formation produces devices or systems appropriate for the incorporation of drugs and various small bioactive molecules [41]. Low pH level (pH less than 6.5) potentiates the antimicrobial activities of chitosan due to the cationic amino groups. Hence, it can easily interact with anionic surfaces of bacterial cells, disrupting the bacterial cell walls and creating leakage of intracellular materials [42]. However, with its low mechanical strength and limited flexibility, chitosan cannot be employed alone in cutting-edge applications. Several approaches have been used to overcome the above-mentioned shortcomings, improve its mechanical properties, and widen its applications, such as blending, crosslinking and grafting with other polymers [43]. One common approach is to blend the free carboxyl groups available in other polymers and their derivatives together with the positively charged amino groups of chitosan, thereby producing a polyelectrolyte complex with improved mechanical stability and strength. Conversely, polymers containing aldehyde groups are frequently used as potential crosslinkers since they easily react with polymers containing amino groups through Schiff’s base reaction. Polysaccharides that can be easily modified to aldehyde functional groups, include cellulose [44], starch [45,46], alginate [47], dextran [48], carboxymethyl cellulose [49], xanthan gum [50], hyaluronic acid [51,52], and pullulan [53].

### 4.2. Pullulan

**Figure 1:**
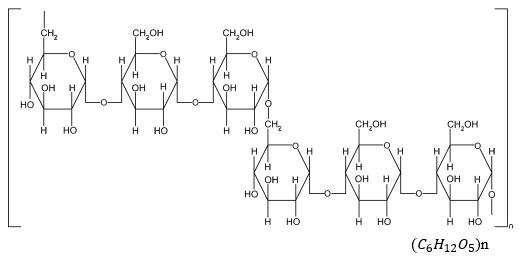
Chemical structure of pullulan.

Pullulan is one of the most fascinating polysaccharides of interest in the pharmaceutical and biomedical fields. The formation of different coexisting glycosidic links means that it possesses unique physical and chemical properties [54,55]. Pullulan is derived from *Aureobasidium pullulans*, a polymorphic fungus [56], and its structure is composed of maltotriose units that are bonded to one another by α-1,6-glycosidic linkages, as shown in Figure 1. It is a homopolysaccharide which is highly soluble in water, less toxic, biodegradable, and nonimmunogenic [57]. The biopolymer is produced through a fermentation process that uses simple sugars as feedstocks. Pullulan is quickly emerging as an important source of polysaccharide and is gradually becoming economically competitive with other polysaccharides, such as natural gums derived from marine algae and other plants. It is easily modified to new derivatives of interest that possess different properties [53]. The US Food and Drug Administration (FDA) has classified pullulan as Generally Regarded as Safe (GRAS) [58]. Therefore, it is used by food industries (such as food processing and packaging), cosmetic industries, and in biomedical applications [59,60,61,62]. Pullulan exhibits significant antiviral, antitumor [63], and antibacterial activities [58,64,65], as well as anticoagulant [66], antithrombotic, and anti-inflammatory properties [67]. Pullulan can be used to incorporate silver nanoparticles, which are powerful antimicrobial agents [68], antimicrobial nanomaterials, and essential oils [69,70,71]. However, considering the toxic nature of metal nanoparticles, a green approach called biosynthesis of metal nanoparticles has been employed. The biosynthesis of silver nanoparticles in pullulan solution is a promising pathway due to its improved solubility, non-toxicity, and compatibility [58]. Nanocomposite films based on pullulan have been reported as fascinating antimicrobial agents against various pathogens [58]. Hassan et al. developed a pullulan hydrogel film incorporated with nisin, lauric alginate, and thymol. It demonstrated excellent properties that can be used as an effective antimicrobial agent against pathogens such as *Staphylococcus aureus*, *E. coli*, *Listeria monocytogenes*, and *Salmonella* spp. [71]. The nanocomposite films could be used as wound dressing materials against wound infections that are caused by multidrug resistance bacteria.

## 5. Oxidative Modifications of Pullulan

Although pullulan possesses valuable properties, it also has some drawbacks, such as poor mechanical properties and high cost [55], which limit its applications in pharmaceutical and biomedical fields. Its valuable properties are therefore enhanced by blending it with other polymers. However, pullulan structure consists of hydroxyl groups liable to chemical modifications. The replacement of hydroxyl group with other functional groups, such as aldehyde and carboxyl groups, can help enhance its performance by improving its mechanical properties via physical or chemical bonds. However, chemically crosslinked hydrogels often exhibit strong interactions and stable networks. Further functionalization of pullulan not only helps to improve its physical and chemical properties, it also widens its biomedical applications [72].

Pullulan can be easily modified to develop new derivatives of interest with different structures and properties. Different approaches have been employed to obtain oxidized derivatives of pullulan, such as periodate and TEMPO oxidations [73,74]. Periodate oxidation of the polymer was first reported by Bruneel et al. in 1993 [75]. Dialdehyde modified pullulan is highly reactive, non-toxic, and more suitable as a crosslinking agent than the commonly used glutaraldehyde crosslinker, which is toxic. TEMPO (2,2,6,6-Tetra methyl piperidinyloxy) modification of pullulan was first reported in 1996 [76] when a large number of carboxyl groups on the homopolysaccharide were synthesized. The reaction has been thoroughly investigated in terms of the properties of the solution [77] as the TEMPO-modified pullulan can be efficiently applied both in the facile green synthesis of silver [78] and in the formation of polyvinyl alcohol hydrogels to improve the network formation [79].

### 5.1. Pullulan Oxidation Using TEMPO

Duceac et al. reported the synthesis of 6-carboxypullulan by chemical oxidation of pullulan with TEMPO. Five grams of pullulan were dissolved in a beaker containing 150 mL of distilled water and stirred for 2 h. Then, 0.12 g of TEMPO and 0.82 g of NaBr were measured and added into the beaker which contained the pullulan solution and were stirred vigorously at a speed of 800 rpm. Later, 100 mL of 8% NaClO solution was poured into the reaction mixture. The pH of the reaction solution was maintained at 10 and carefully monitored in order to maintain an oxidative environment with optimal pH level. The reaction was stopped after 4 h by adding ethylene glycol, and a large volume of acetone was poured into the reaction mixture, which precipitated the oxidized product. The obtained product was washed, dialyzed, and subsequently lyophilized [53].

### 5.2. Aldehyde Modification of Pullulan

**Figure 2:**
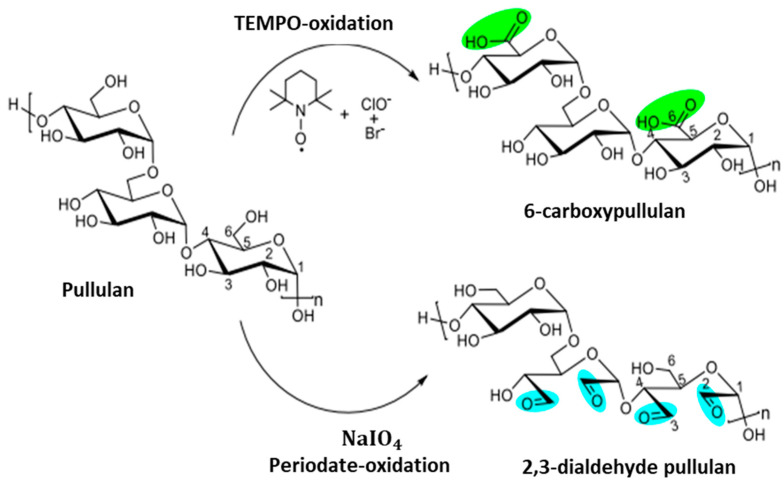
Illustration of pullulan oxidation using TEMPO and periodate. Reproduced from [53], with permission from Elsevier, 2022.

Chemical oxidation of pullulan to dialdehyde has been reported using periodate. Using distilled water, 1 % *w/v* of the pullulan solution was prepared at room temperature. Then, 2.63 g of sodium periodate were added to the pullulan solution. Reaction was allowed for 6 h at room temperature while the mixture was magnetically stirred. The reaction mixture was sealed with aluminum foil to prevent light exposure. The aldehyde modified pullulan, as shown in Figure 2, was precipitated using large amounts of acetone, then dialyzed and freeze-dried [53].

TEMPO and periodate oxidations of pullulan have been investigated to improve the mechanical property and performance of pullulan on its own and in combination with other polymers [80]. Aldehyde modified pullulan hydrogels and its composites with other polymers, such as gelatin, have been investigated to enhance the mechanical strength of hydrogel formulations due to the crosslinking of aldehyde groups present in the oxidized pullulan and the amino group in gelatin, which form a strong covalent bond [81]. Pullulan derivatives can also be used as hydrogels stabilizing agents on modification through succinylation, urethane derivatization, and modification of cholesterol. Pullulan derivatives can also promote antimicrobial activity. Succinyl pullulan crosslinked with carboxymethyl chitosan has been investigated for its ability to boost antimicrobial activity in the wound healing process [82].

## 6. Applications of Pullulan-Based Biomaterials as Wound Dressings and Skin Tissue Engineering Scaffolds

Currently, pullulan composites with different biopolymers, such as chitosan, chitin, gelatin, and collagen, have gained considerable importance and have been used to develop films, sponges, and hydrogels for wound dressings, skin tissue scaffolds, and drug delivery devices. Considering the beneficial properties of pullulan and other polymers, such as chitosan, synthesis of hydrogel composites using these polysaccharides will greatly enhance wound healing.

**Figure 3:**
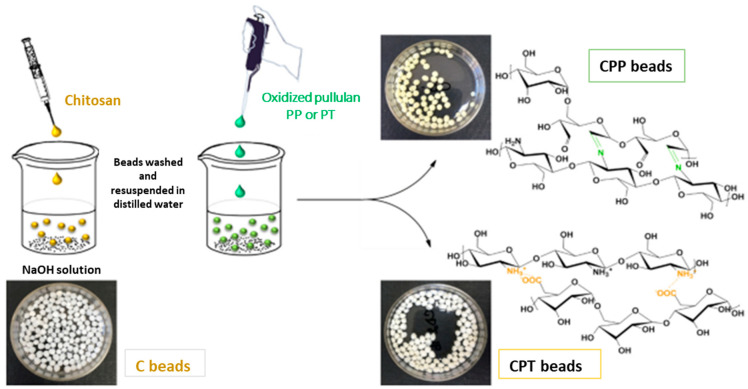
Preparation of chitosan and oxidized pullulans. Reproduced from [53], with permission from Elsevier, 2022. CPP: chitosan/pullulan periodate; CPT: chitosan/pullulan TEMPO.

Duceac et al. recently fabricated a chitosan-pullulan composite with tunable pore size and targeted properties for drug delivery applications. The fabricated composite structures consisted of a core of chitosan covered with different forms of modified pullulans, that is, one contained carboxyl groups and the other contained aldehyde groups. The researchers demonstrated that the two types of materials produced possessed different physical and biological properties [53]. The chitosan-TEMPO oxidized pullulan beads were formed by physical bonds, while the chitosan-periodate oxidized pullulan beads were produced by chemical linkage. The researchers demonstrated that the two different composites had high antibacterial activities. Hemocompatibility studies of the composites showed mild coagulation as a result of low amount of free amino acid groups on the surface of the chitosan composites; this occurred because the amino groups are involved in ionic or covalent interactions with the carboxyl or aldehyde oxidized pullulan. The hemostatic property of a material is a characteristic regarding the biological activity and its applications. It is very important for fabricated wound dressings to show hemostatic actions. Their findings showed that these composites could not only act as drug delivery devices but also as modern wound dressings with excellent properties [53]. Figure 3 illustrates the two chemical pathways of chitosan/oxidized pullulan hydrogel beads.

**Figure 4:**
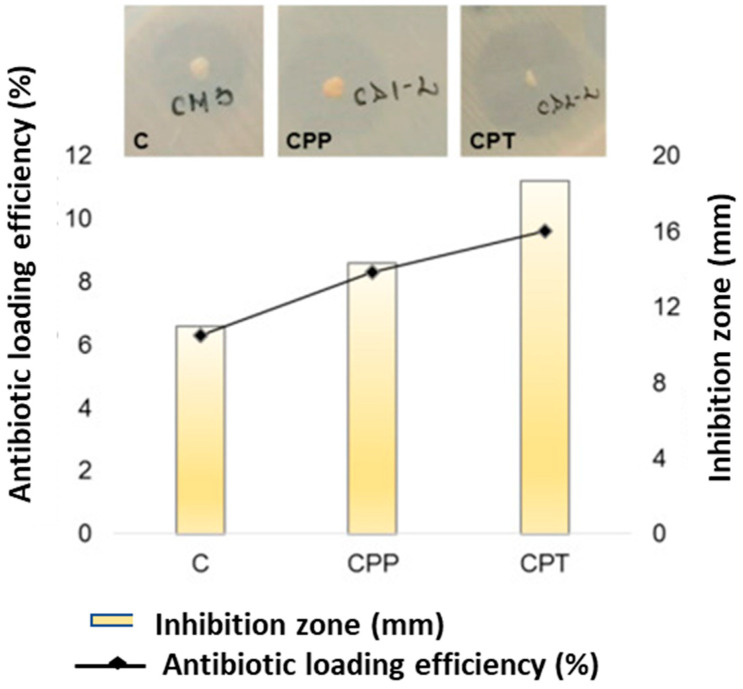
Antibiotic loading efficiency and antibacterial activity of the beads. Reproduced from [53], with permission from Elsevier, 2022. C: chitosan; CPP: chitosan/pullulan periodate; CPT: chitosan/pullulan TEMPO.

In addition, they observed that TEMPO-oxidized beads (CPT) had the best bactericidal activity, which could be explained by the higher antibiotic incorporation in their network. The obtained results proved that drug incorporated beads exhibited antibacterial activity. The hydrogel beads showed distinct inhibition area, which confirmed the drug release and antibacterial activity against *Staphylococcus aureus*. The functionalized beads, CP and CPT, had higher inhibition zones (14.33 mm and 18.66 mm, respectively), and chitosan beads had the smallest inhibition zone (11 mm) (Figure 4). Their findings demonstrated that surface functionalization of pullulan led to higher drug encapsulation efficiencies.

**Figure 5:**
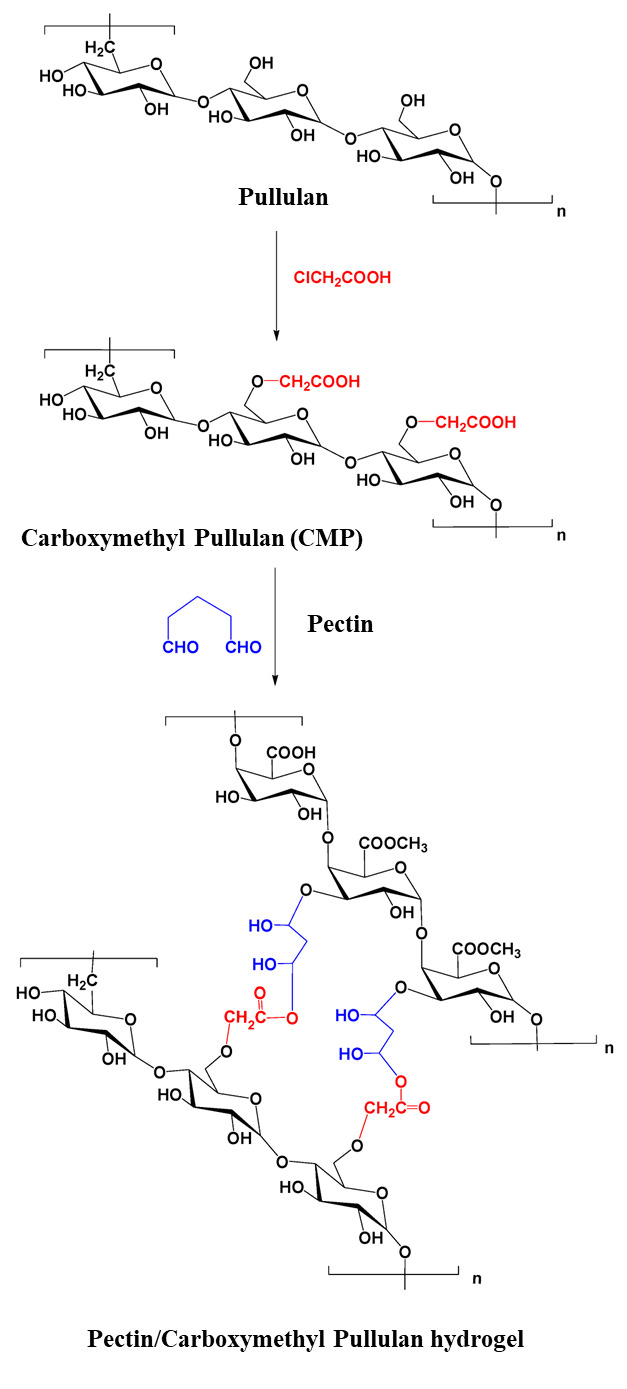
Preparation of pectin/carboxymethyl pullulan hydrogel. Reproduced from [83], with permission from MDPI, 2022.

Emam et al. synthesized polyvinylpyrrolidone (povidine)-bound iodine (PI) loaded in pectin/carboxymethyl pullulan hydrogel. Carboxymethyl pullulan was first prepared through etherification reaction in an alkaline pH of aqueous/organic solution. Pullulan was suspended in isopropanol and 1M NaOH was added dropwise; it was then magnetically stirred for 60 min, followed by the dropwise addition of monochloroacetic acid in the reaction mixture at 70 °C for 5 h. Synthesized carboxymethyl pullulan was crosslinked with pectin, with glutaraldehyde used as the crosslinker, to obtain pectin-carboxymethyl pullulan hydrogel. Polyvinylpyrrolidone (povidine)-bound iodine (PI) acted as an antiseptic reagent against skin infections and wound healing. It was demonstrated that the release of PI from the hydrogel matrix was highly efficient as a result of good swelling ability of the composite network. The hydrogel could be used as a wound dressing for treating skin injuries and as a drug delivery device [83]. Figure 5 illustrates the synthetic pathway of pectin/carboxymethyl pullulan hydrogel.

**Figure 6:**
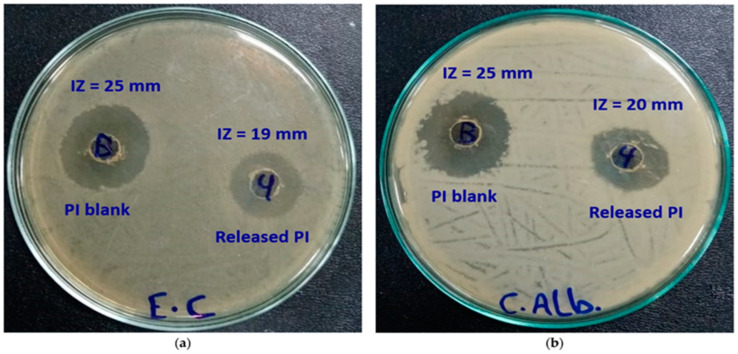
Antimicrobial activity of pure PI (left) and released PI (right) against (**a**) *Escherichia coli* (**b**) *Candida albicans*. Reproduced from [83], with permission from MDPI, 2023. PI: Polyvinylpyrrolidone (povidine)-bound iodine.

The antimicrobial activities of the synthesized pectin/carboxymethyl pullulan hydrogel were investigated, including the release of polyvinylpyrrolidone (povidine)-bound iodine (PI), against two pathogens, *Escherichia coli* and *Candida albicans.* It was confirmed that the biological activity of released PI from the hydrogel was highly effective. Studies showed that the inhibition areas of released PI from the hydrogel samples were 19 mm and 20 mm for *Escherichia coli* and *Candida albicans*, respectively (Figure 6) [83]. In addition, the antimicrobial activity of pure PI was 25 mm for both pathogens. The low inhibition zones of the released PI could be related to the concentration of PI in the released hydrogel samples. However, the activity of PI was not affected after it was released, except the low PI concentration, which led to decreased activity in the inhibition zone.

**Figure 7:**
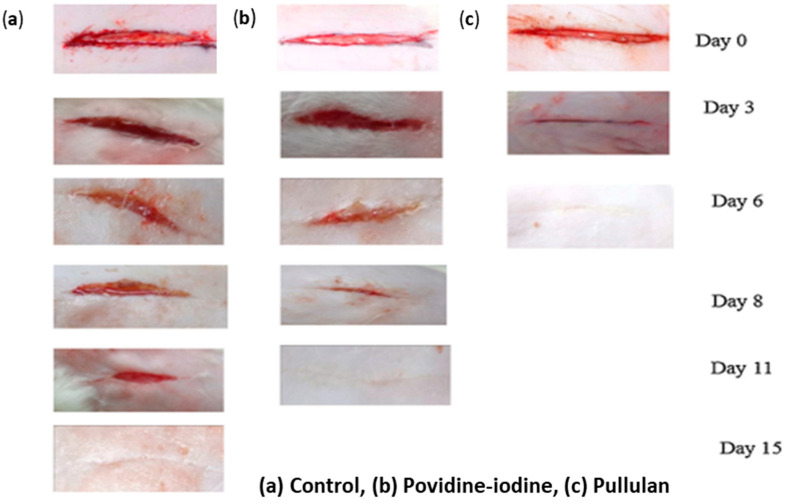
Wound healing rate in sutureless wounds in rats. Reproduced from [84], with permission from Elsevier, 2022.

Priya et al. synthesized 10% pullulan hydrogel with no crosslinkers and evaluated its wound healing efficiency in daily topical administration. Their findings showed faster healing of wounds when the hydrogel was administered topically. They explained that the fast healing was due to the controlled release and availability of the therapeutic agent at the wound site as well as the antioxidant and energy generating properties of pullulan. Pullulan, being a biodegradable polysaccharide polymer, could also be a source of energy for cells, such as fibroblasts, which are actively involved in the healing process. Furthermore, the increases in the rate of wound closure and the decreases in the healing time with pullulan treated wounds could result from its hydroscopic nature, which facilitated bacterial dehydration, inactivated them, and reduced their surface area. Dehydration of wound fluid may improve cells and tissues oxygenation, promoting wound healing. Their histological examination demonstrated improved growth of fibroblasts and epithelialization in wounds treated with pullulan [84]. This clearly supports pullulan as a potent material for wound healing. Figure 7 shows that incision wounds treated with pullulan healed completely after six days while povidine-iodine treated wounds healed after eleven days.

**Figure 8:**
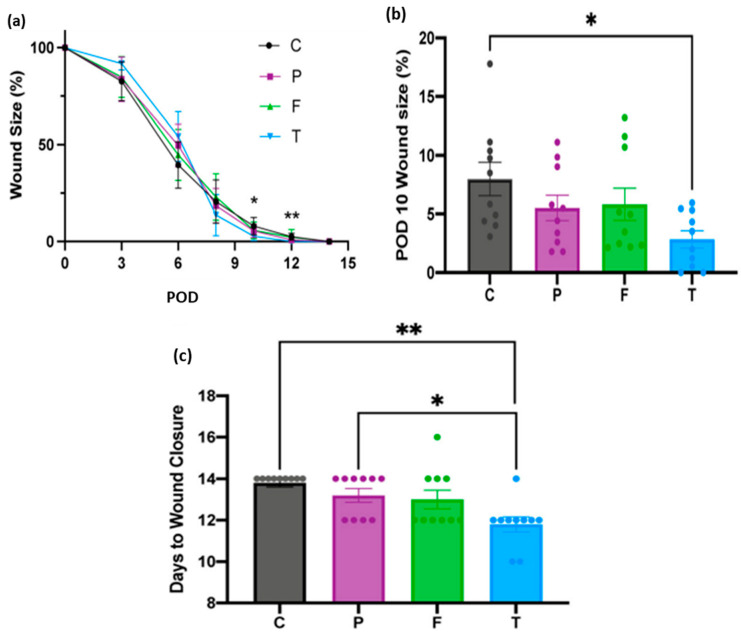
(**a**) Measurement of wound area by treatment group (**b**) Wound size at POD 10 (**c**) Days until total wound closure by treatment group (* *p* < 0.05, ** *p* < 0.01). C = control, P = Promogran™, F = Fibracol^®^ Plus, and T = TauTona Wound Dressing (TWD), Pullulan/collagen dressing. POD = Postoperative day. Reproduced from [85], with permission from Wiley 2022.

Chen et al. fabricated a pullulan/collagen hydrogel with tunable, suitable biomechanical properties and improved biocompatibility for wound treatment and regeneration. In this study, they compared the efficacy of the synthesized hydrogel with two marketed wound dressings, Promogran™ (55% collagen and 45% oxidized regenerated cellulose) and Fibracol ^®^ Plus (90% collagen and 10% alginate) [85]. They used a mouse excisional wound model and dressed the wounds with the commercial dressings and the synthesized pullulan/collagen dressing (TauTona wound dressing, TWD) alongside untreated control wounds, then investigated the healing process. Their findings demonstrated that pullulan/collagen hydrogel dressings enhanced collagen architecture and alignment and accelerated healing in murine wounds after 14 days compared to the other commercial dressings. The measurement of the wound area over time is presented in Figure 8. At postoperative days, PODs 10 and 12, the area of the wound treated with the synthesized hydrogel was significantly reduced compared to control wounds (Figure 8a). The sizes of wounds treated with Promogran™ and Fibracol^®^ Plus were not significantly different from the pullulan/collagen dressing. The percentage of the wound sizes at PODs 10, as illustrated in Figure 8b, demonstrated that the wounds treated with pullulan/collagen dressing had smaller wound size than Promogran™ and Fibracol^®^ Plus. They further proved that pullulan/collagen treated wounds demonstrated a significant decrease in macrophages, lymphocytes, and overall tissue response, which accelerated wound repair compared to Promogran ™. Finally, their studies showed that stromal cells derived from adipose tissues seeded in the developed hydrogel promoted healing in murine burn model, reduced time of wound closure, decreased scaring and developed collagen network [85,86]. The pullulan/collagen hydrogel demonstrated clinical feasibility and ease of use. Recently, pullulan/collagen hydrogel dressing has been manufactured by the TauTona group in Redwood City, Canada, and has been referred to as TauTona wound dressing (TWD) [85].

Nicholas et al. investigated the efficacy of pullulan/gelatin scaffolds on skin regeneration. They fabricated a cost-effective pullulan/gelatin hydrogel with suitable mechanical properties for skin substitutes and cells, such as fibroblasts and keratinocytes, that were grown in the hydrogel. The excisional wounds treated with hydrogels exhibited less macrophage infiltration, decreased inflammation, and improved angiogenesis after 14 days of post mouse-skin biopsy compared to the control. Their findings suggested that the pullulan/gelatin hydrogel could be suitable in skin wounds with a high level of inflammation, such as chronic wounds and burns [87].

Biomedical sponges are soft and flexible materials with a highly interconnected porous network. The high swelling rate of these scaffolds and fast hemostatic ability can make them suitable for preventing the accumulation of unwanted wound fluids. In addition, sponges with high water content provide a moist wound environment and protect it from bacterial infection. Wang et al., developed succinyl pullulan/carboxymethyl chitosan sponges as a potential wound dressing. Succinyl pullulan (pullulan-COOH) was prepared by mixing succinic anhydride and an aqueous solution of pullulan. Succinyl pullulan and carboxymethyl chitosan were mixed and crosslinked with 1-ethyl-3-(3-dimethylami nopropyl)-carbodiimide/N-hydroxy succinimide (EDC/NHS) and the sponges were obtained. The crosslinker introduced amide bonds between the carboxyl groups present in pullulan-COOH and amino groups in carboxymethyl chitosan, which has been confirmed to be non-toxic. They demonstrated that the sponges maintained a good, moist environment that significantly reduced the wound area. Histological evaluation revealed that the sponges promoted proliferation of the fibroblast and improved epithelialization [82]. Further wound dressing materials based on pullulan are summarized in Table 3.

Over the past few years, pullulan has been reported to have applications in vascular engineering, bone tissue engineering [100], and skin tissue repairs. Tissue engineering is a recently growing field that assists in the regeneration and repair of injured tissues and potentiates patients’ wound healing process. Hydrogel as a skin substitute greatly depends on the material from which it is developed. The non-toxic, nonimmunogenic, non-mutagenic, and antioxidant properties of pullulan have shown it to be a suitable material for skin regeneration applications. Pullulan methacrylate hydrogels have promising potentials in the production of cell-laden microscale tissues to incorporate cells in a three-dimensional environment [53,101]. Research has shown that cells encapsulated in pullulan methacrylate hydrogel possessed excellent viability, proliferation, and accelerated the repair of wounds in rats and mice [102]. Pullulan/collagen hydrogels can be used as skin scaffolds to accelerate wound healing due to their excellent mechanical properties, such as porosity and pore size [103,104]. These hydrogel scaffolds can easily replicate skin architecture and promote encapsulation of stem cells and elements of wound healing for the restoration of skin tissues.

Pullulan scaffolds have demonstrated potential antioxidant properties which can be of great importance for skin regeneration. The antioxidant property protects the stromal cells from oxidative damage [54]. Atila et al. reported a 3D electrospun pullulan-cellulose acetate scaffold which had excellent cytocompatibility, as the cells could easily adhere, spread, and grow on the hydrogel scaffolds. As such these scaffolds had great potential for skin tissue engineering applications [105]. Pullulan significantly promoted cell proliferation and enhanced cell adhesion. Therefore, pullulan and its composites could be potent materials in skin tissue engineering applications.

**Figure 9:**
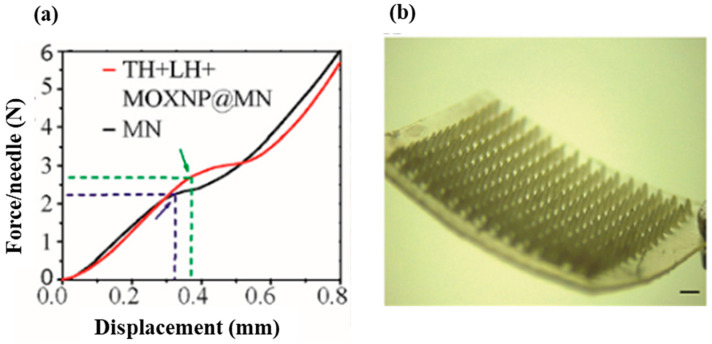
(**a**) Mechanical strength of blank microneedle (MN) and Thrombin (TH) + Lidocaine (LH) + Moxifloxacin nanoparticles (MOXNP@MN). (**b**) Visual presentation of pullulan-based microneedles (Magnification: 8×, scale bar: 2 mm). Reproduced from [106], with permission from Elsevier, 2023.

Recently, Younas et al. developed a multifunctional pullulan microneedle patch loaded with chitosan/fucoidan nanoparticles for differential release of moxifloxacin, lidocaine, and thrombin. Chitosan and fucoidan were used to synthesize moxifloxacin nanoparticles with a diameter of 258.0 ± 10.86 nm and surface charge 45.1 ± 3.9 mV. Lidocaine (LH), thrombin (TH), and moxifloxacin nanoparticles (MOXNP) were then encapsulated in a 30% (*w*/*w*) pullulan-based microneedle patch. Their findings demonstrated that the microneedle patch achieved rapid hemostasis/analgesia and sustained antibacterial activity. The patch facilitated the rapid release of thrombin and could offer efficient coagulation. Their results proved that the pullulan microneedle patch was highly biocompatible with combined hemostatic, analgesic, and prolonged antibacterial effects. Therefore, the multifunctional patch based on polysaccharides (pullulan, chitosan, and fucoidan) can be used for high-quality wound healing [106]. The researchers investigated the mechanical strength of the pullulan-based microneedles and claimed that the microneedles both with and without drug encapsulation exhibited outstanding mechanical properties. The blank microneedle (MN) and the drug loaded sample had significant displacement at 2.27 N/needle and 2.73 N/needle, respectively (Figure 9). In addition, they reported that the combined polysaccharides developed microneedle patch had high biocompatibility. Transdermal drug delivery is a modern delivery system for therapeutic agents possessing systemic side effects. Pullulan-based dissolving microneedles have been utilized for transdermal delivery of small and large bioactive molecules [107].

**Figure 10:**
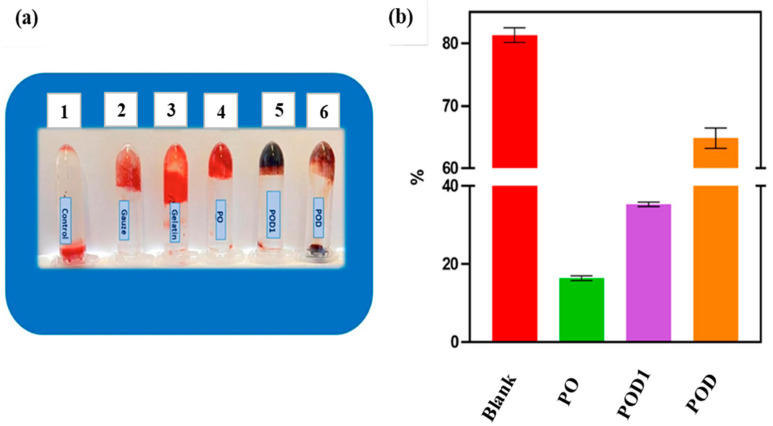
Results of in vitro blood coagulation: (**a**) Photographs of blood clotting. 1: control; 2: medical gauze; 3: gelatin; 4: PO (oxidized pullulan); 5: POD (oxidized pullulan/dopamine cryogel prepared by in situ loading of dopamine; 6: POD1 (oxidized pullulan/dopamine cryogel sample prepared by post-incorporation of dopamine); (**b**) Blood clotting index of the cryogel samples incubated in whole blood for 10 min. Reproduced from [93] with permission from MDPI, 2023.

The hemocompatibility of pullulan is one of the important criteria for its applications in skin tissue engineering and wound management. Baron and co-workers fabricated a hemostatic wound dressing based on dialdehyde pullulan and dopamine. The developed multifunctional cryogels were prepared by a series of combinations of hemi(acetal) and Schiff base interactions. The assessment of hemostatic effect was performed based on the blood clotting index (BCI). They prepared three different samples of cryogels. The first cryogel sample was based solely on dialdehyde oxidized pullulan (PO). The two dialdehyde oxidized pullulan/dopamine cryogels were prepared based on the mechanism of dopamine incorporation. The first oxidized pullulan/dopamine cryogel sample was fabricated by in situ loading of dopamine followed by lyophilization (POD), and the second oxidized pullulan/dopamine sample was obtained by post-incorporation of dopamine (POD1). The obtained results demonstrated that BCI values of the oxidized pullulan (PO) and oxidized pullulan/dopamine (POD1) hydrogel samples were <50 % (Figure 10) which was attributed to better blood clotting ability of the hydrogels. In addition, they observed lower blood clotting indices of the cryogels with increased oxidation of pullulan [108]. Hence, hemostatic wound dressings can help to reduce blood loss in chronic and acute wounds and fasten wound healing [93,97,108]. They further observed that periodate-oxidation pullulan could form stable hydrogels due to the hemi(acetal) interactions, and also that dopamine interacted with the aldehyde groups, resulting in improved mechanical stability of the hydrogels networks. Therefore, hemostatic activity and mechanical stability of pullulan-based hydrogels suggested that they can be promising materials for wound dressings and skin tissue scaffolds.

## 7. Conclusions

The choice of material is an important factor to be considered when designing an ideal wound dressing. Natural polysaccharides are considered to be ideal wound dressing materials due to their biocompatibility, biodegradability, and eco-friendly properties. Here, we overviewed the chemical modifications and properties of wound dressing materials based on natural homopolysaccharide pullulan and using other natural biopolymers, such as chitosan and gelatin. We reviewed their applications in wound dressing and skin tissue scaffolds, and their application as drug delivery devices. In the past few decades, pullulan hydrogels have achieved enormous attention due to their special properties. The beneficial properties of pullulan result from its glycosidic bond. It has therefore occupied a niche area in biomedical and pharmaceutical fields. Pullulan and chitosan have unique properties; they have high-water retention and are biocompatible, biodegradable, antimicrobial, and non-toxic, among others. Such properties warrant further investigation of pullulan-based hydrogels in combination with other biomaterials for the development of enhanced multifunctional antimicrobial, antioxidant, anti-inflammatory, smart wound dressings, and drug delivery devices. In contrast to its important biological and physicochemical properties, pullulan has some drawbacks such, as poor mechanical properties and high cost. However, these properties can be enhanced by combining pullulan with other polymers. In addition, pullulan derivatives are still under investigation and have not yet been approved for commercial use. Therefore, investigations are required to produce pullulan derivatives with suitable properties to improve their applications in wound healing and tissue engineering. Hence, more pullulan-based hydrogels wound dressings with excellent performance and improved mechanical properties, and which are both multifunctional and cost-effective, need to be developed in the near future.

## Figures and Tables

**Table 1:** Types of wound dressings.

Category	Benefit	Examples	Reference
Passive wound dressing	Mainly protect the wound from the external surroundings	Gauze, bandages and plasters	[28]
Allografts (skin substitutes)	Accelerate wound closure and replace the skin function, thereby boosting healing	Allografts, tissue-engineered derivatives, autografts	[29]
Bioactive natural dressing	Have antimicrobial, antioxidant, bio-adhesive, and proliferative properties	Collagen, chitosan, alginate, chitin	[30]
Artificial materials	Enhance wound healing and offer barriers against bacterial infections	Sprays, hydrocolloids, films, foams and gels	[31]

**Table 2:** Summary of the properties of ideal wound dressings.

Property	Description
Maintain wound moisture	Prevents wound from drying
Excellent gas transmission	Allows the exchange of oxygen between the wound and the environment
Superabsorbent capabilities	Removes excess exudates
Protect against microbial contaminations	Possesses antimicrobial properties
Eco-friendly	Biodegradable
Excellent wound healing regulator	Reduces inflammation, stimulates release of growth factors, tissue regeneration, and prevents scaring
Provide mechanical protection	Acts as a physical barrier to prevent further damage to the wound
Stop bleeding	Possesses excellent homeostatic properties to prevent further blood loss
Adhesiveness	Possesses easy and comfortable removal properties
Nonimmunogenic and biocompatible	Is non-allergic and non-toxic to the body
Cost-effective and commercially available	Improves patients’ compliance through accessibility and affordability

**Table 3:** Additional pullulan-based hydrogels wound dressings.

Application	System Used	Drug/Growth Enhancing Factor(s)	Cell Types Treated	Reference
Wound healing	Pullulan film	-	Rat skin cells	[88]
Wound dressing	Keratin/pullulan/PVA hydrogel membrane	Cefotaxime	Incision on male SD rat skin cells	[89]
Wound dressing and antibacterial effect	Carboxymethyl pullulan hydrogels	Gentamicin	In vitro drug release studies in phosphate buffer saline solution	[90]
Wound healing	Hyaluronic/pullulan/ grafted-Pluronic F127 hydrogel	Curcumin	Female rat skin cells	[91]
High oxidative stress wound dressing	Pullulan hydrogels	Mesenchymal stromal cell	Anterior and posterior full thickness transverse incisions on skin cells	[92]
Wound dressing	Pullulan/dopamine hydrogel	-	In vitro studies of sheep blood	[93]
Wound healing	Pullulan/chitosan composite nanofibers	Tannic acid	NIH 3T3 mouse embryonic fibroblast cells	[94]
Wound dressing and antioxidant effect	Pullulan/bacterial cellulose hydrogel	Vitamin C and E	In vitro studies in phosphate saline buffer	[95]
Early cutaneous wound healing	Pullulan/collagen composite hydrogels	-	Full thickness skin cells incisions	[96]
Wound dressing	Collagen/pullulan hydrogel	Polydatin	Wistar rat skin cells	[97]
Wound healing	Cholesterol bearing pullulan nanogels	Prostaglandin E1	Full thickness defect on rat dermal cells	[98]
Wound dressing and drug delivery system	Pu-g-p(AA-co-IA) hydrogel film	Ampicillin	In vitro drug release study in phosphate buffer saline solution	[99]

Pu-g-p(AA-co-IA) = Pullulan grafted poly(acrylic acid-co-itaconic acid); PVA = Polyvinyl alcohol.

## Data Availability

Not applicable.

## References

[1] Firlar I., Altunbek M., McCarthy C., Ramalingam M., Camci-Unal G. (2022). Functional Hydrogels for Treatment of Chronic Wounds. Gels.

[2] Turner N.J., Badylak S.F. (2015). The use of biologic scaffolds in the treatment of chronic nonhealing wounds. Adv. Wound Care.

[3] Sivaraj D., Chen K., Chattopadhyay A., Henn D., Wu W., Noishiki C., Magbual N.J., Mittal S., Mermin-Bunnell A.M., Bonham C.A. (2021). Hydrogel scaffolds to deliver cell therapies for wound healing. Front. Bioeng. Biotechnol..

[4] Naseri-Nosar M., Ziora Z.M. (2018). Wound Dressings from Naturally occurring Polymers: A Review on Homopolysac charide-based Composites. Carbohydr. Polym..

[5] Archana D., Dutta J., Dutta P.K. (2013). Evaluation of chitosan nano dressing for wound healing: Characterization, in vitro and in vivo studies. Int. J. Biol. Macromol..

[6] Farzamfar S., Naseri-Nosar M., Samadian H., Mahakizadeh S., Tajerian R., Rahmati M., Vaez A., Salehi M. (2018). Taurine-loaded poly (ε-caprolactone)/gelatin electrospun film as a potential wound dressing material: In vitro and in vivo evaluation. J. Bioact. Compat. Polym..

[7] Savencu I., Iurian S., Porfire A., Bogdan C., Tomuța I. (2021). Review of advances in polymeric wound dressing films. React. Funct. Polym..

[8] Bai M.Y., Chen M.C., Yu W.C., Lin J.Y. (2017). Foam dressing incorporating herbal extract: An all-natural dressing for potential use in wound healing. J. Bioact. Compat. Polym..

[9] Mano J., Silva G., Azevedo H.S., Malafaya P., Sousa R., Silva S., Boesel L., Oliveira J.M., Santos T., Marques A. (2007). Natural origin biodegradable systems in tissue engineering and regenerative medicine: Present status and some moving trends. J. R. Soc. Interface.

[10] Mogoşanu G.D., Grumezescu A.M. (2014). Natural and synthetic polymers for wounds and burns dressing. Int. J. Pharm..

[11] Jain A., Gupta Y., Jain S.K. (2007). Perspectives of biodegradable natural polysaccharides for site specific drug delivery to the colon. J. Pharm. Pharm. Sci..

[12] d’Ayala G.G., Malinconico M., Laurienzo P. (2008). Marine derived polysaccharides for biomedical applications: Chemical modification approaches. Molecules.

[13] Malafaya P.B., Silva G.A., Reis R.L. (2007). Natural–origin polymers as carriers and scaffolds for biomolecules and cell delivery in tissue engineering applications. Adv. Drug Deliv. Rev..

[14] Cui R., Zhang L., Ou R., Xu Y., Xu L., Zhan X.-Y., Li D. (2022). Polysaccharide-Based Hydrogels for Wound Dressing: Design Considerations and Clinical Applications. Front. Bioeng. Biotechnol..

[15] Tang N., Zhang R., Zheng Y., Wang J., Khatib M., Jiang X., Zhou C., Omar R., Saliba W., Wu W. (2022). Highly Efficient Self-Healing Multifunctional Dressing with Antibacterial Activity for Sutureless Wound Closure and Infected Wound Monitoring. Adv. Mater..

[16] Yu H.C., Zhang H., Ren K., Ying Z., Zhu F., Qian J., Ji J., Wu Z.L., Zheng Q. (2018). Ultrathin κ-Carrageenan/Chitosan Hydrogel Films with High Toughness and Antiadhesion Property. ACS Appl. Mater. Inter..

[17] Harding K.G., Jones V., Price P. (2000). Topical Treatment: Which Dressing to Choose. Diabetes Metab. Res. Rev..

[18] Moura L.I.F., Dias A.M.A., Carvalho E., de Sousa H.C. (2013). Recent Advances on the Development of Wound Dressings for Diabetic Foot Ulcer Treatment-A Review. Acta Biomater..

[19] Kibungu C., Kondiah P.P.D., Kumar P., Choonara Y.E. (2021). This Review Recent Advances in Chitosan and Alginate-Based Hydrogels for Wound Healing Application. Front. Mater..

[20] Line A.S., Morykwas M.J., Line S.W. (1995). Use of Cultured Human Epidermal Xenografts for Wound Treatment in Nonhuman Primates. J. Zoo Wildl. Med..

[21] Greenwood J.E., Dearman B.L. (2012). Split Skin Graft Application over an Integrating, Biodegradable Temporizing Polymer Matrix. J. Burn Care Res..

[22] Morgan D. (2002). Wounds—What Should a Dressing Formulary Include?. Hosp. Pharm..

[23] Dhivya S., Padmab V.V., Santhini E. (2015). Wound dressings–a review. BioMedicine.

[24] Morgado P.I., Lisboa P.F., Ribeiro M.P., Miguel S.P., Simões P.C., Correia I.J., Aguiar Ricardo A. (2014). Poly (vinyl alcohol)/chitosan asymmetrical membranes: Highly controlled morphology toward the ideal wound dressing. J. Membrane Sci..

[25] Heilmann S., Kuchler S., Wischke C., Lendlein A., Stein C., Schafer-Korting M. (2013). A thermosensitive morphine-containing hydrogel for the treatment of large-scale skin wounds. Int. J. Pharm..

[26] Kong M.S., Koh W.-G., Lee H.J. (2022). Controlled Release of Epidermal Growth Factor from Furfuryl-Gelatin Hydrogel Using *in Situ* Visible Light-Induced Crosslinking and Its Effects on Fibroblasts Proliferation and Migration. Gels.

[27] Thönes S., Rother S., Wippold T., Blaszkiewicz J., Balamurugan K., Moeller S., Ruiz-Gómez G., Schnabelrauch M., Scharnweber D., Saalbach A. (2019). Hyaluronan/collagen hydrogels containing sulfated hyaluronan improve wound healing by sustained release of Heparin-Binding EGF-like growth factor. Acta Biomater..

[28] Qu J., Zhao X., Liang Y., Zhang T., Ma P.X., Guo B. (2018). Antibacterial Adhesive Injectable Hydrogels with Rapid Self-Healing, Extensibility and Compressibility as Wound Dressing for Joints Skin Wound Healing. Biomaterials.

[29] Bradford C., Freeman R., Percival S.L. (2009). In Vitro study of Sustained Antimicrobial Activity of a New Silver Alginate Dressing. J. Am. Coll. Certif. Wound Spéc..

[30] Almeida J.F., Ferreira P., Lopes A., Gil M.H. (2011). Photocrosslinkable Biodegradable Responsive Hydrogels as Drug Delivery Systems. Int. J. Biol. Macromol..

[31] Liang M., Chen Z., Wang F., Liu L., Wei R., Zhang M. (2019). Preparation of Self-regulating/anti-adhesive Hydrogels and Their Ability to Promote Healing in Burn Wounds. J. Biomed. Mater. Res. Part B Appl. Biomater..

[32] Silva R., Singh R., Sarker B., Papageorgiou D.G., Juhasz-Bortuzzo J.A., Roether J.A., Cicha I., Kaschta J., Schubert D.W., Chrissafis K. (2018). Hydrogel Matrices Based on Elastin and Alginate for Tissue Engineering Applications. Int. J. Biol. Macromol..

[33] Zhu T., Mao J., Cheng Y., Liu H., Lv L., Ge M., Li S., Huang J., Chen Z., Li H. (2019). Recent Progress of Polysaccharide-Based Hydrogel Interfaces for Wound Healing and Tissue Engineering. Adv. Mater. Inter..

[34] Lee K.Y., Mooney D.J. (2012). Alginate: Properties and Biomedical Applications. Prog. Polym. Sci..

[35] Becker A., Klapczynski A., Kuch N., Arpino F., Simon-Keller K., De la Torre C., Sticht C., van Abeelen F.A., Oversluizen G., Gretz N. (2016). Gene Expression Profiling Reveals Aryl Hydrocarbon Receptor as a Possible Target for Photobiomodulation when Using Blue Light. Sci. Rep..

[36] Panyamao P., Ruksiriwanich W., Sirisa-ard P., Charumanee S. (2020). Injectable Thermosensitive Chitosan/Pullulan-Based Hydrogels with Improved Mechanical Properties and Swelling Capacity. Polymers.

[37] Lu Y., Fan L., Yang L.Y., Huang F., Ouyang X. (2020). PEI-modified core-shell/bead-like amino silica enhanced poly (vinyl alcohol)/chitosan for diclofenac sodium efficient adsorption. Carbohydr. Polym..

[38] Tan Y., Wu H., Xie T., Chen L., Hu S., Tian H., Wang Y., Wang J. (2020). Characterization and antibacterial effect of quaternized chitosan anchored cellulose beads. Int. J. Biol. Macromol..

[39] Gupta A., Kowalczuk M., Heaselgrave W., Britland S.T., Martin C., Radecka I. (2019). The Production and Application of Hydrogels for Wound Management: A Review. Eur. Polym. J..

[40] Güiza-Argüello V.R., Solarte-David V.A., Pinzón-Mora A.V., Ávila-Quiroga J.E., Becerra-Bayona S.M. (2022). Current Advances in the Development of Hydrogel-Based Wound Dressings for Diabetic Foot Ulcer Treatment. Polymers.

[41] Bagre A.P., Jain K., Jain N.K. (2013). Alginate coated chitosan core shell nanoparticles for oral delivery of enoxaparin: In vitro and in vivo assessment. Int. J. Pharm..

[42] Hosseinnejad M., Jafari S.M. (2016). Evaluation of different factors affecting antimicrobial properties of chitosan. Int. J. Biol. Macromol..

[43] Qu B., Luo Y. (2020). Chitosan-based hydrogel beads: Preparations, modifications and appli cations in food and agriculture sectors—A review. Int. J. Biol. Macromol..

[44] Kim U.J., Lee Y.R., Kang T.H., Choi J.W., Kimura S., Wada M. (2017). Protein adsorption of dialdehyde cellulose-crosslinked chitosan with high amino group contents. Carbohydr. Polym..

[45] Zuo Y., Liu W., Xiao J., Zhao X., Zhu Y., Wu Y. (2017). Preparation and characterization of dialdehyde starch by one-step acid hydrolysis and oxidation. Int. J. Biol. Macromol..

[46] Yong H., Bai R., Bi F., Liu J., Qin Y., Liu J. (2020). Synthesis, characterization, antioxidant and antimicrobial activities of starch aldehyde-quercetin conjugate. Int. J. Biol. Macromol..

[47] Balakrishnan B., Joshi N., Jayakrishnan A., Banerjee R. (2014). Self-crosslinked oxidized alginate /gelatin hydrogel as injectable, adhesive biomimetic scaffolds for cartilage regeneration. Acta Biomater..

[48] Flynn J., Durack E., Collins M.N., Hudson S.P. (2020). Tuning the strength and swelling of an injectable polysaccharide hydrogel and the subsequent release of a broad spectrum bacteriocin, nisin A. J. Mater. Chem. B.

[49] Rahman M.S., Hasan M.S., Nitai A.S., Nam S., Karmakar A.K., Ahsan M.S., Shiddiky M.J.A., Ahmed M.B. (2021). Recent Developments of Carboxymethyl Cellulose. Polymers.

[50] Guo J., Ge L., Li X., Mu C., Li D. (2014). Periodate oxidation of xanthan gum and its crosslinking effects on gelatin-based edible films. Food Hydrocoll..

[51] Pandit A.H., Mazumdar N., Ahmad S. (2019). Periodate oxidized hyaluronic acid-based hydrogel scaffolds for tissue engineering applications. Int. J. Biol. Macromol..

[52] Valachova K., Svik K., Biro C., Collins M.N., Jurcik R., Ondruska L., Soltes L. (2020). Impact of ergothioneine, hercynine, and histidine on oxidative degradation of hyaluronan and wound healing. Polymers.

[53] Duceac A.I., Vereștiuc L., Coroaba A., Arotăriței D., Coseri S. (2021). All-polysaccharide hydrogels for drug delivery applications: Tunable chitosan beads surfaces via physical or chemical interactions, using oxidized pullulan. Int. J. Biol. Macromol..

[54] Singh R.S., Kaur N., Rana V., Kennedy J.K. (2016). Recent insights on applications of pullulan in tissue engineering. Carbohydr. Polym..

[55] Li X., Zhao S., Chen L., Zhou Q., Qiu J., Xin X., Zhang Y., Yuan W., Tian C., Yu X. (2023). High-level production of pullulan from high concentration of glucose by mutagenesis and adaptive laboratory evolution of Aureobasidium pullulans. Carbohydr. Polym..

[56] Feng Z., Chen S., Ahmad A., Chen L., Bai W. (2022). Ultra-high molecular weight pullulan-based material with high deformability and shape-memory properties. Carbohydr. Polym..

[57] Singh R.S., Kaur N., Rana V., Kennedy J.K. (2017). Pullulan: A novel molecule for biomedical applications. Carbohydr. Polym..

[58] Rai M., Wypij M., Ingle A.P., Trzcinska-Wencel J., Golinska P. (2021). Emerging Trends in Pullulan-Based Antimicrobial Systems for Various Applications. Int. J. Mol. Sci..

[59] Luís A., Ramos A., Domingues F. (2020). Pullulan Films Containing Rockrose Essential Oil for Potential Food Packaging Applications. Antibiotics.

[60] Krasniewska K., Pobiega K., Gniewosz M. (2019). Pullulan—Biopolymer with Potential for Use as Food Packaging. Int. J. Food Eng..

[61] Coltelli M.-B., Danti S., De Clerck K., Lazzeri A., Morganti P. (2020). Pullulan for Advanced Sustainable Body- and Skin-Contact Applications. J. Funct. Biomater..

[62] Luís A., Ramos A., Domingues F. (2021). Pullulan–Apple Fiber Biocomposite Films: Optical, Mechanical, Barrier, Antioxidant and Antibacterial Properties. Polymers.

[63] Emam H.E., Ahmed H.B. (2020). Antitumor/antiviral carbon quantum dots based on carrageenan and pullulan. Int. J. Biol. Macromol..

[64] Li S., Yi J., Yu X., Wang Z., Wang L. (2020). Preparation and characterization of pullulan derivative/chitosan composite film for potential antimicrobial applications. Int. J. Biol. Macromol..

[65] Soto K., Hernández-Iturriaga M., Loarca-Piña G., Luna-Barcenas G., Mendoza S. (2019). Antimicrobial effect of nisin electrospun amaranth: Pullulan nanofibers in apple juice and fresh cheese. Int. J. Food Microbiol..

[66] Hamidi M., Okoro V.O., Milan B.P., Khalili R.M., Samadian H., Nie L., Shavandi A. (2022). Fungal exopolysaccharides: Properties, sources, modifications, and biomedical applications. Carbohydr. Polym..

[67] Tabasum S., Noreen A., Maqsood M.F., Umar H., Akram N., Nazli Z.-i-H., Chatha S.A.S., Zia K.M. (2018). Review on versatile applications of blends and composites of pullulan with natural and synthetic polymers. Int. J. Biol. Macromol..

[68] Lee J.H., Jeong D., Kanmani P. (2019). Study on physical and mechanical properties of the biopolymer/silver based active nanocomposite films with antimicrobial activity. Carbohydr. Polym..

[69] Gniewosz M., Krasniewska K., Woreta M., Kosakowska O. (2013). Antimicrobial Activity of a Pullulan-Caraway Essential Oil Coating on Reduction of Food Microorganisms and Quality in Fresh Baby Carrot. J. Food Sci..

[70] Pinto R., Almeida A., Fernandes S.C., Freire C., Silvestre A., Neto C.P., Trindade T. (2013). Antifungal activity of transparent nanocomposite thin films of pullulan and silver against Aspergillus niger. Colloids Surf. B Biointerfaces.

[71] Hassan A.H., Cutter C.N. (2020). Development and evaluation of pullulan-based composite antimicrobial films (CAF) incorporated with nisin, thymol and lauric arginate to reduce foodborne pathogens associated with muscle foods. Int. J. Food Microbiol..

[72] Hernandez-Tenorio F., Giraldo-Estrada C. (2022). Characterization and chemical modification of pullulan produced from a submerged culture of Aureobasidium pullulans ATCC 15233. Polym. Test..

[73] Zhang L., Liu J., Zheng X., Zhang A., Zhang X., Tang K. (2019). Pullulan dialdehyde crosslinked gelatin hydrogels with high strength for biomedical applications. Carbohydr. Polym..

[74] Coseri S., Bercea M., Harabagiu V., Budtova T. (2016). Oxidation vs. degradation in polysaccharides: Pullulan—A case study. Eur. Polym. J..

[75] Bruneel D., Schacht E. (1993). Chemical modification of pullulan: Periodate oxidation. Polymer.

[76] De Nooy A.E.J., Besemer A.C., Van Bekkum H., Van Dijk J.A.P.P., Smit J.A.M. (1996). TEMPO-mediated oxidation of pullulan and influence of ionic strength and linear charge density on the dimensions of the obtained polyelectrolyte chains. Macromolecules.

[77] Spatareanu A., Bercea M., Budtova T., Harabagiu V., Sacarescu L., Coseri S. (2014). Synthesis, characterization and solution behaviour of oxidized pullulan. Carbohydr. Polym..

[78] Coseri S., Spatareanu A., Sacarescu L., Rimbu C., Suteu D., Spirk S., Harabagiu V. (2015). Green synthesis of the silver nanoparticles mediated by pullulan and 6-carboxypullulan. Carbohydr. Polym..

[79] Baron R.I., Bercea M., Avadanei M., Lisa G., Biliuta G., Coseri S. (2019). Green route for the fabrication of self-healable hydrogels based on tricarboxy cellulose and poly(vinyl alcohol). Int. J. Biol. Macromol..

[80] Agrawal S., Budhwani D., Gurjar P., Telange D., Lambole V. (2022). Pullulan based derivatives: Synthesis, enhanced physicochemical properties, and applications. Drug Deliv..

[81] Wang Y., Guo Z., Qian Y., Zhang Z., Lyu L., Wang Y., Ye F. (2019). Study on the electrospinning of gelatin/pullulan composite nanofibers. Polymers.

[82] Wang X., Zhang D., Wang J., Tang R., Wei B., Jiang Q. (2017). Succinyl Pullulan-Crosslinked Carboxymethyl Chitosan Sponges for Potential Wound Dressing. Int. J. Polym. Mater. Polym..

[83] Emam H.E., Mohamed A.L. (2021). Controllable Release of Povidone-Iodine from Networked Pectin@Carboxymethyl Pullulan Hydrogel. Polymers.

[84] Priya V.S., Iyappan K., Gayathri V.S., William S., Suguna L. (2016). Influence of pullulan hydrogel on sutureless wound healing in rats. Wound Med..

[85] Chen K., Sivaraj D., Davitt M.F., Leeolou M.C., Henn D., Steele S.R., Huskins S.L., Trotsyuk A.A., Kussie H.C., Greco A.H. (2022). Pullulan-Collagen hydrogel wound dressing promotes dermal remodelling and wound healing compared to commercially available collagen dressings. Wound Rep. Reg..

[86] Barrera J.A., Trotsyuk A.A., Maan Z.N., Bonham C.A., Larson M.M.R., Mittermiller P.A., Henn D., Chen K., Mays M.C.J., Mittal M.S. (2021). Adipose-derived stromal cells seeded in pullulan-collagen hydrogels improve healing in murine burns. Tissue Eng. Part A.

[87] Nicholas M.N., Jeschke M.G., Amini-Nik S. (2016). Cellularized bilayer pullulan-gelatin hydrogel for skin regeneration. Tissue Eng. Part A.

[88] Suguna L. (2014). Wound healing potential of a biodegradable film from pullulan in rats. Clin. Exp. Dermatol..

[89] Khaliq T., Sohail M., Shah S.A., Mahmood A., Kousar M., Jabeen N. (2022). Bioactive and multifunctional keratin-pullulan based hydrogel membranes facilitate re-epithelization in diabetic model. Int. J. Biol. Macromol. Part B.

[90] Li H., Yang J., Hu X., Liang J., Fan Y., Zhang X. (2011). Superabsorbent polysaccharide hydrogels based on pullulan derivate as antibacterial release wound dressing. J. Biomed. Mater. Res..

[91] Shah S.A., Sohail M., Minhas M.U., Khan S., Hussain Z., Mahmood A., Kousar M., Thu H.E., Abbasi M., Kashif M.U.R. (2021). Curcumin-laden hyaluronic acid-co-Pullulan-based biomaterials as a potential platform to synergistically enhance the diabetic wound repair. Int. J. Biol. Macromol..

[92] Wong V.W., Rustad K.C., Glotzbach J.P., Sorkin M., Inayathullah M., Major M.R., Longaker M.T., Rajadas J., Gurtner G.C. (2011). Pullulan hydrogels improve mesenchymal stem cell delivery into high-oxidative-stress wounds. Macromol. Biosci..

[93] Baron R.I., Duceac I.A., Morariu S., Bostanaru-Iliescu A.-C., Coseri S. (2022). Hemostatic Cryogels Based on Oxidized Pullulan/Dopamine with Potential Use as Wound Dressings. Gels.

[94] Xu F., Weng B., Gilkerson R., Materon L.A., Lozano K. (2015). Development of tannic acid/chitosan/pullulan composite nanofibers from aqueous solution for potential applications as wound dressing. Carbohydr. Polym..

[95] Atila D., Karataş A., Keskin D., Tezcaner A. (2022). Pullulan hydrogel-immobilized bacterial cellulose membranes with dual-release of vitamin C and E for wound dressing applications. Int. J. Biol. Macromol..

[96] Wong V.W., Rustad K.C., Galvez M.G., Neofytou E., Glotzbach J.P., Januszyk M., Major M.R., Sorkin M., Longaker M.T., Rajadas J. (2011). Engineered pullulan-collagen composite dermal hydrogels improve early cutaneous wound healing. Tissue Eng. Part A.

[97] Selvakumar G., Lonchin S. (2022). Bioactive functional collagen-oxidized pullulan scaffold loaded with polydatin for treating chronic wounds. Biomater. Adv..

[98] Kobayashi H., Katakura O., Morimoto N., Akiyoshi K., Kasugai S. (2009). Effects of cholesterol-bearing pullulan (CHP)-nanogels in combination with prostaglandin E1 on wound healing. J. Biomed. Mater. Res. Part B Appl. Biomater. J..

[99] Mert H., Ozkahraman B., Damar H. (2020). A novel wound dressing material: Pullulan grafted copolymer hydrogel via UV copolymerization and crosslinking. J. Drug Deliv. Sci. Technol..

[100] Omar N.A., Amédée J., Letourneur D., Fricain J.-C., Fenelon M. (2022). Recent Advances of Pullulan and/or Dextran-Based Materials for Bone Tissue Engineering Strategies in Preclinical Studies: A Systematic Review. Front. Bioeng. Biotechnol..

[101] Bae H., Ahari A.F., Shin H., Nichol J.W., Hutson C.B., Masaeli M., Kim S.H., Aubin H., Yamanlar S., Khademhosseini A. (2011). Cell-laden microengineered pullulan methacrylate hydrogels promote cell proliferation and 3D cluster formation. Soft Matter.

[102] Fricain J.C., Schlaubitz S., Le Visage C., Arnault I., Derkaoui S.M., Siadous R., Catros S., Lalande C., Bareille R., Renard M. (2013). A nano-hydroxyapatite-pullulan/dextran polysaccharide composite macroporous material for bone tissue engineering. Biomaterials.

[103] Galvez M.G., Wong V.W., Chang E.I., Major M., Carre L., Kandimalla R., Bhatt K.A., Rajadas J., Longaker M.T., Gurtner G.C. (2009). Pullulan-collagen hydrogel scaffold as a dermal substitute. J. Am. Coll. Surg..

[104] Iswariya S., Bhanu Keerthi A.V., Velswamy P., Uma T.S., Perumal P.T. (2016). Design and development of a piscine collagen blended pullulan hydrogel for skin tissue engineering. RSC Adv..

[105] Atila D., Keskin D., Tezcaner A. (2015). Cellulose acetate based 3-dimensional electrospun scaffolds for skin tissue engineering applications. Carbohydr. Polym..

[106] Younas A., Dong Z., Hou Z., Asad M., Li M., Zhang N. (2023). A chitosan/fucoidan nanoparticle-loaded pullulan microneedle patch for differential drug release to promote wound healing. Carbohydr. Polym..

[107] Singh S.R., Kaur N., Singh D., Purewal S.S., Kennedy F.J. (2023). Pullulan in pharmaceutical and cosmeceutical formulations: A review. Int. J. Biol. Macromol..

[108] Zheng W., Zhang Z., Li Y., Wang L., Fu F., Diao H., Liu X. (2022). A novel pullulan oxidation approach to preparing a shape memory sponge with rapid reaction capability for massive hemorrhage. Chem. Eng. J..

